# Papillary muscle native T_1_ time is associated with severity of functional mitral regurgitation in patients with non-ischemic dilated cardiomyopathy

**DOI:** 10.1186/1532-429X-18-S1-P244

**Published:** 2016-01-27

**Authors:** Shingo Kato, Sébastien Roujol, Shadi Akhtari, Francesca N Delling, Jihye Jang, Tamer Basha, Sophie Berg, Kraig V Kissinger, Beth Goddu, Warren J Manning, Reza Nezafat

**Affiliations:** 1 Beth Israel Deaconess Medical Center, Boston, MA USA; 2 Yokohama City University, Yokohama, Japan

## Background

Functional mitral regurgitation is one of the important complications of non-ischemic dilated cardiomyopathy (DCM). Non-contrast native T_1_ mapping enables non-invasive quantification of myocardial fibrosis. We sought to assess the potential relationship between papillary muscle T_1_ time and severity of mitral regurgitation in DCM patients.

## Methods

Forty DCM patients (55 ± 13 years) and 20 healthy adult control subjects (54 ± 13 years) were enrolled. Slice interleaved T_1_ mapping sequence (STONE) was employed for the native T_1_ mapping, which enables acquisition of 5 slices in the short-axis plane within a 90 sec free-breathing scan. For calculating papillary muscle native T_1_ time, both anterior and posterior papillary muscles were manually traced on custom software (MediaCare, Boston, MA, USA). We measured papillary muscle diameter, length and shortening on cine MRI. Phase contrast images were acquired perpendicular to the proximal ascending aorta to quantify blood flow. Mitral regurgitation volume was calculated as the difference between the LV stroke volume and ascending aorta forward flow. DCM patients were allocated into 2 groups based on the presence or absence of functional mitral regurgitation.

## Results

Papillary muscle T_1_ time was significantly elevated in DCM patients with mitral regurgitation (n = 22) in comparison to those without mitral regurgitation (n = 18) (anterior papillary muscle: 1127 ± 36 msec vs 1063 ± 16 msec, p < 0.001; posterior papillary muscle: 1124 ± 30 msec vs 1062 ± 19 msec, p < 0.001), but LV T_1_ time was similar (1129 ± 38 msec vs 1134 ± 58 msec, p = 0.93). Multivariate linear regression analysis showed that papillary muscle native T_1_ time (β=0.109, 95%CI: 0.048-0.170, p = 0.001) and tenting height (β=1.334, 95%CI: 0.434-2.234, p = 0.005) are correlated with mitral regurgitant fraction (Table 1). Elevated papillary muscle T_1_ time was associated with larger diameter, longer length and decreased papillary muscle shortening (all p values <0.05).

**Table 1:** Determinants for Severity of Functional Mitral Regurgitation in Non-ischemic Dilated Cardiomyopathy

	β	SE	95% CI for β	P-value
LVEDVI, mL/m2	-0.006	0.027	-0.061 - 0.049	0.82
LVEF,%	-0.009	0.099	-0.211 - 0.193	0.93
Mitral annulus (4 chamber), mm	0.170	0.151	-0.138 - 0.479	0.27
Tenting height, mm	1.334	0.440	0.434 - 2.234	0.005
Papillary muscle T1 time	0.109	0.030	0.048 - 0.170	0.001

CI, confidence interval; DCM, dilated cardiomyopathy; LVEDVI, left ventricular end-diastolic volume index; LVEF, left ventricular ejection fraction; MR, mitral regurgitation; SE, standard error

## Conclusions

In DCM, papillary muscle native T_1_ time is significantly elevated and related to mitral regurgitant fraction. These results suggest that papillary muscle diffuse fibrosis might be associated with severity of functional mitral regurgitation in this population.

